# Case Report: Telitacicept for interstitial lung disease associated with idiopathic inflammatory myopathies: an observational study

**DOI:** 10.3389/fphar.2025.1647543

**Published:** 2025-11-14

**Authors:** Xue Chen, Mengshan Li, MingFang Sun, XinTong Xie, Xi Zhao, Yan Chen, HuanZi Dai

**Affiliations:** Department of Rheumatology and Immunology, Daping Hospital, Army Medical University, Chongqing, China

**Keywords:** idiopathic inflammatory myopathies, interstitial lung disease, B-lymphocyte stimulator, rituximab, telitacicept

## Abstract

**Background:**

Interstitial lung disease (ILD) is the most common complication and the major cause of mortality among patients with idiopathic inflammatory myopathies (IIMs). Currently, no recommended standard treatment for IIM-associated ILD. In this observational study, we evaluated the efficacy and safety of telitacicept in treating IIM-associated ILD.

**Methods:**

We included 10 patients with IIM-associated ILD; of them, seven had antisynthetase syndrome-associated ILD, one had anti-MDA5 antibody-positive dermatomyositis (DM)-associated ILD, and 2 had DM-associated ILD. Four patients with severe ILD were treated with a combination of rituximab (RTX) (375 mg/m^2^/week for 4 weeks) and telitacicept (160 mg/week). Six patients had refractory IIM-associated ILD; of them, two received RTX (375 mg/m^2^/week for 4 weeks) in combination with telitacicept (160 mg/week), and four were treated with telitacicept (160 mg/week) alone because they had an increased infection risk.

**Result:**

Over the 24-week follow-up, glucocorticoid dosage was reduced to 5–10 mg/day and that of telitacicept treatment was increased to 160 mg every 2 weeks in all patients. These patients exhibited alleviation of rash, joint swelling and pain, muscle pain and weakness, and dyspnea. Compared with before treatment, the Manual Muscle Testing 8 score and PaO2/FiO2 ratio increased by 25.1% and 28.2% after treatment, respectively. Lung function also exhibited considerable improvements, with percentages of forced vital capacity and diffusing capacity of the lungs for carbon monoxide increasing by 20.4% and 30.2%, respectively. Posttreatment chest high-resolution computed tomography revealed significant improvements compared with baseline. Only one patient experienced a mild lung infection, and no further infections occurred after telitacicept dose was reduced. One patient was administered additional nintedanib for pulmonary fibrosis due to insufficient improvement in lung function.

**Conclusion:**

Telitacicept demonstrates substantial clinical efficacy in the treatment of IIM-associated ILD, accompanied by a low infection rate and a favorable safety profile.

## Graphical Abstract

**Figure:**
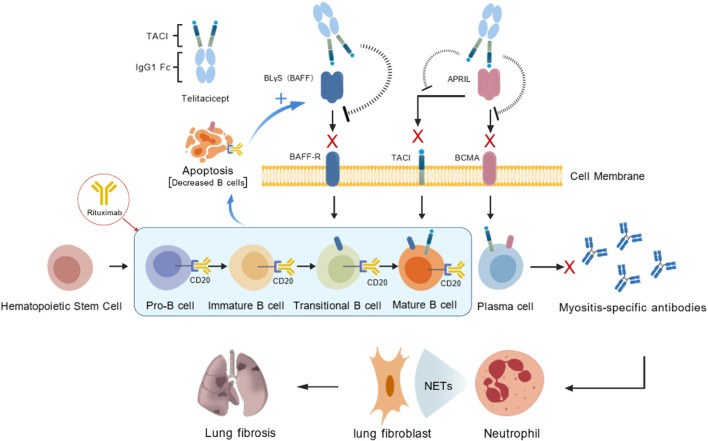

## 1 Introduction

Idiopathic inflammatory myopathies (IIMs) are a group of autoimmune diseases primarily involving skeletal muscles of the limbs and the skin (Lundberg et al., 2017). The clinical manifestations of IIMs are diverse and highly heterogeneous. They can be classified into several subtypes, including dermatomyositis (DM), antisynthetase syndrome (ASS), immune-mediated necrotizing myopathy (IMNM), polymyositis (PM), and sporadic inclusion body myositis (sIBM); of them, DM, ASS, and IMNM are the most prevalent (Lundberg et al., 2017; Tanboon and Nishino, 2019).

Interstitial lung disease (ILD) is the most prevalent complication associated with IIMs and the leading cause of hospitalization and mortality in patients with IIMs. Based on the onset pattern of ILD, ILD can be categorized into acute/subacute and chronic types. Patients with acute/subacute ILD exhibit a lower survival rate than those with chronic ILD (Fujisawa, 2021; Vu et al., 2023).

Thus far, no standard treatment has been recommended for IIM-associated ILD (IIM-ILD). Research on supporting optimal treatment options for IIM-ILD has been limited; most relevant studies have used a retrospective nonrandomized observational or case series design. Treatment approaches primarily include glucocorticoids (GCs), immunosuppressants [e.g., cyclophosphamide (CTX), mycophenolate mofetil (MMF), calcineurin inhibitors (CNIs), and azathioprine], rituximab (RTX), tofacitinib, and plasma exchange (Fujisawa, 2021; Thong et al., 2023). In this study, we assessed the outcomes of telitacicept treatment in 10 IIM-ILD cases. Our results may offer a novel therapeutic option for IIM-ILD management.

## 2 Materials and methods

### 2.1 Study design

This was a retrospective observational study evaluating the efficacy of telitacicept, alone or in combination with RTX, in IIM-ILD treatment. This study spanned 24 weeks and included a cohort of 10 patients.

The study design, methods, objectives, and plans were thoroughly reviewed and approved by our institutional ethics committee to ensure ethical integrity. In accordance with ethical research standards, all participants were asked to provide informed consent after we offered a comprehensive explanation of the study’s purpose, methodology, and potential implications for patients.

### 2.2 Inclusion and exclusion criteria

We included patients who were diagnosed as having IIMs according to the 2020 European Neuromuscular Center (ENMC) diagnostic criteria for DM or the 2011 Solomon classification criteria for ASS, had a chest high-resolution computed tomography (HRCT) scan indicating the presence of ILD, and developed progressive ILD after traditional treatments (e.g., GCs and immunosuppressants). If a patient was newly diagnosed, ILD was required to have presented severely, characterized by a PaO2/FiO2 ratio of <300 or the presence of respiratory failure.

We excluded patients who had severe infections, drug allergies, or experienced serious adverse reactions, or patients who had not signed the informed consent form.

### 2.3 Data collection

Before commencing treatment, we collected baseline clinical data of the included patients, including age, sex, body mass index, and history of previous treatments. Subsequently, at 0, 12, and 24 weeks after treatment initiation, we collected the following patient data: arterial blood gas analysis, 6-min walk test (6MWT), Manual Muscle Testing 8 (MMT-8) scores, lung function assessments, chest HRCT scans, and laboratory indicators [namely, erythrocyte sedimentation rate (ESR), C-reactive protein (CRP), ferritin, creatine kinase (CK), alanine aminotransferase (ALT), aspartate aminotransferase (AST), lactate dehydrogenase (LDH), and immunoglobulin G (IgG)].

### 2.4 Observational outcomes

#### 2.4.1 Efficacy outcomes

We evaluated improvements in IIM and ILD symptoms in the included patients after 24 weeks of treatment. Simultaneously, we compared the changes in the PaO2/FiO2 ratio, forced vital capacity percentage (FVC%), diffusing capacity of the lungs for carbon monoxide percentage (DLCO%), and MMT-8 score at baseline and 12 and 24 weeks after treatment initiation.

#### 2.4.2 Safety outcomes

We evaluated the incidence rates of adverse drug reactions, including allergies, infections, hypogammaglobulinemia, leukopenia, gastrointestinal disorders, and neurological disorders.

### 2.5 Statistical analysis

All statistical analyses were performed using SPSS (version 26). We used *t* tests to compare pretreatment and posttreatment data. A *p*-value of <0.05 was considered to indicate statistical significance.

## 3 Results

Among the 10 patients included in this study, four were male and six were female; their ages ranged from 45 to 75 years. Of them, seven patients had ASS-associated ILD, one had anti-MDA5 antibody-positive DM-associated ILD, and two had DM-associated ILD. Table 1 presents the baseline data of all patients.

**TABLE 1:** Patient baseline data and laboratory indicators, PaO2/FiO2, 6MWT, and MMT8 before and after treatment. All laboratory indicators returned to normal after treatment.

Case number	Age/Sex	BMI	ARS	MDA5	Comorbiditie/Reason for treatment	Treatment	Observation period	CK (40-200 U/L)	LDH (120-250 U/L)	ALT (7-40 U/L)	AST (13-35 U/L)	CRP (0-8 mg/L)	ESR (0-20 mm/h)	Ferritin (11-306 ng/ml)	IgG (8.7-17 g/L)	PaO2/FiO2	6MWT (m)	MMT8	Adverse effects
1	51/F	19.5	JO-1	-	urinary tract infection Invalid treatment for MMF	Prednisone acetate Telitacicept	Baseline	70.7	211	15.8	23.7	1.06	29	412	11.99	380	428	150	No adverse effects
							12 W	32.4	142	11.2	16.2	0.5	11	58.93	6.53	409	451	150	
							24 W	39.1	186.5	11.8	16.9	0.5	8	103.37	6.71	433	520	150	
2	45/F	21.9	PL-12	-	High Tuberculosis enzyme-linked immunospot Invalid treatment for CTX	Prednisone acetate Telitacicept	Baseline	387.4	547.5	22.8	43.9	15.78	52	402.8	19.52	395	480	150	No adverse effects
							12 W	41.5	162.8	13.6	14.1	0.5	20	133.3	8.18	426	512	150	
							24 W	51.2	162.1	10.9	15.5	0.5	12	4.63	7.43	458	540	150	
3	47/F	27.4	JO-1	-	Invalid treatment for tacrolimus Pulmonary infection	Prednisone acetate Telitacicept	Baseline	71.3	212.6	15.9	15.9	0.5	37	50.49	15.04	384	552	150	pruritus at the injection site
							12 W	55.8	147.8	19.8	14.4	0.5	9	40.4	8.7	412	568	150	
							24 W	74.1	191.2	23.1	17.8	0.5	13	62.54	8.79	433	576	150	
4	71/M	22.5	-	-	Invalid treatment for CTX Positive hepatitis B surface antigen High Tuberculosis enzyme-linked immunospot	Prednisone acetate Telitacicept Nintedanib	Baseline	11375.3	781.5	160	238.5	14.41	51	362.53	7.46	400	200	75	No adverse effects
							12 W	266.8	384	43.9	27.5	0.5	6	165.97	6.25	426	470	104	
							24 W	196.3	367	31.7	24.2	2.93	5	202.13	6.67	438	485	130	
5	64/F	28.2	PL-12	-	Invalid treatment for MMF	Prednisone acetate RTX Telitacicept	Baseline	32.4	198.1	46.7	42.4	28.81	76.5	233.21	9.49	298	380	150	No adverse effects
							12 W	33.6	264	90.4	57.4	4.51	14	132.19	9.66	323	396	150	
							24 W	35.1	271.4	59.6	40.8	3.42	15	128.93	8.3	352	457	150	
6	56/M	22.2	Jo-1	-	Invalid treatment for MMF, CTX, Tacrolimus	Prednisone acetate RTX Telitacicept	Baseline	4123	417.5	162.2	178.9	31.04	23	1275.16	13.14	285	200	96	Mild pulmonary infection
							12 W	748	277.1	64.7	66.5	10.8	15	407.88	11.47	333	264	108	
							24 W	98.6	105.4	36	39.5	2.6	9	89.5	7.63	372	358	130	
7	75/F	21.5	PL-12	-	Respiratory failure	Prednisone acetate RTX Telitacicept	Baseline	949.1	503.3	34.5	50.3	21.87	71	371.74	24.12	270	360	138	No adverse effects
							12 W	221.5	284	14.6	19	14.86	65	161.28	9.29	333	384	142	
							24 W	151.7	236.2	9.7	16	8.56	23	85.74	7.29	352	420	148	
8	65/M	22.9	JO-1	-	Respiratory failure	Prednisone acetate RTX Telitacicept	Baseline	1093	282.5	178.5	210.9	2.79	15	151.62	19.37	276	312	132	No adverse effects
							12 W	28.4	175.9	17.6	20.6	0.5	3	166.49	5.66	390	396	140	
							24 W	65.3	207.7	33.3	30.5	0.5	6	171.37	6.56	409	432	148	
9	45/M	23.6	-	+	Respiratory failure hoarseness, drinking water and choking	Prednisone acetate RTX Telitacicept	Baseline	656.8	479.4	47.4	92.2	18.22	29	1233.3	14.34	271	288	110	pruritus at the injection site
							12 W	58.6	295.4	25.2	33.3	2.5	16	474.31	7.23	366	437	136	
							24 W	55.6	227.9	10.2	19.8	4.64	23	281.22	7.35	428	495	145	
10	61/F	21.7	-	-	Respiratory failure	Prednisone acetate RTX Telitacicept	Baseline	2005	619.8	102	94.2	7.4	69	121.6	21.37	257	270	128	No adverse effects
							12 W	777	356.9	40.6	45.2	0.71	21	48.26	12.8	356	310	144	
							24 W	69	174	34.2	37	0.43	13	35.26	8.42	448	370	148	

Of all patients, one experienced hoarseness, difficulty drinking water, and choking, in addition to involvement of the skin, muscles, joints, and lungs. Four patients with severe ILD and PaO2/FiO2 ratio <300 were treated with GCs and RTX, followed by telitacicept. Six patients with a poor response to conventional immunosuppressants were classified as having refractory IIM-ILD. Of them, two patients received treatment with RTX combined with telitacicept, whereas four were treated only with telitacicept because they had an increased risk of infection.

**FIGURE 1:**
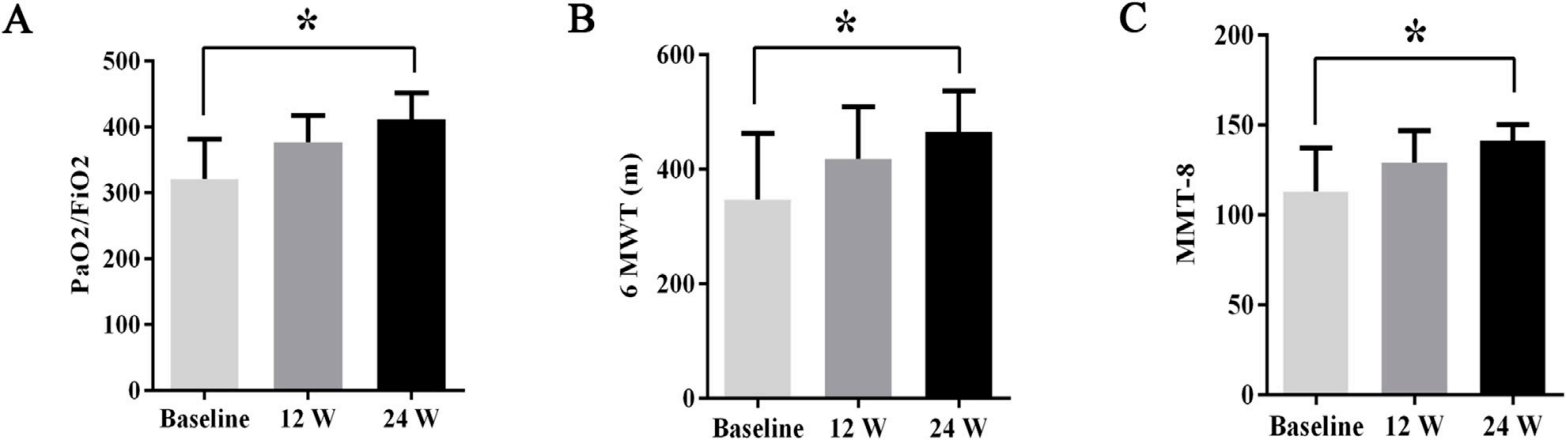
The changes in PaO2/FiO2 ratios, 6MWT outcomes, and MMT8 scores before and after treatment. Post-treatment results demonstrated significant improvements: **(A)** PaO2/FiO2 ratios increased by 28.2%, **(B)** 6MWT outcomes improved by 34%, and **(C)** MMT8 scores increased by 25.1%. All data are presented as means ± standard errors of the means, with *p <0.05 indicating statistical significance compared to pretreatment data.

**FIGURE 2:**
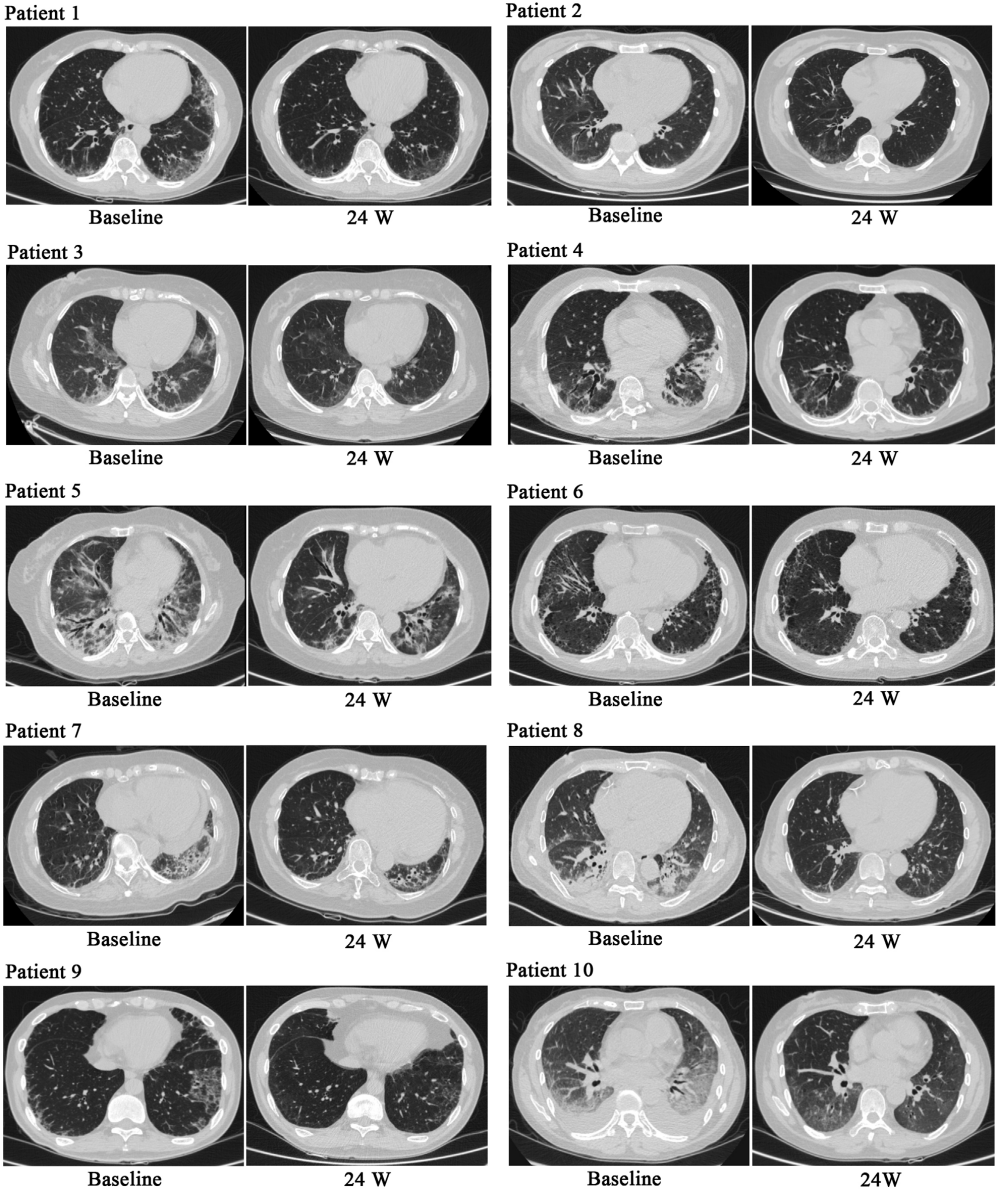
Chest HRCT before and after treatment. After treatment, chest HRCT scans demonstrated significant improvements compared with baseline.

**FIGURE 3:**
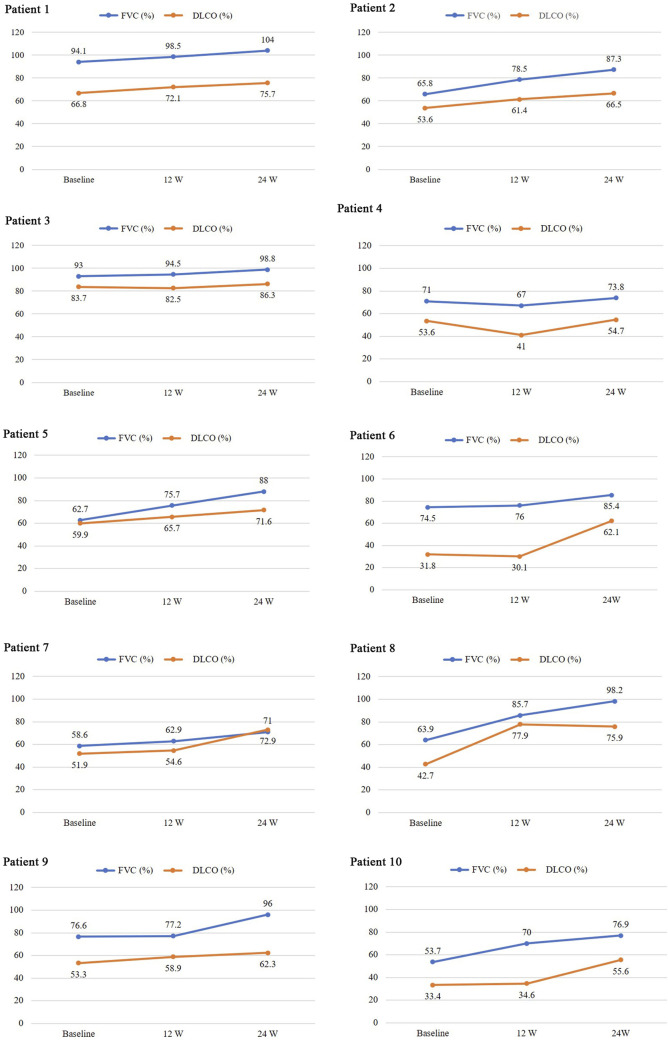
Pulmonary function before and after treatment.

**FIGURE 4:**
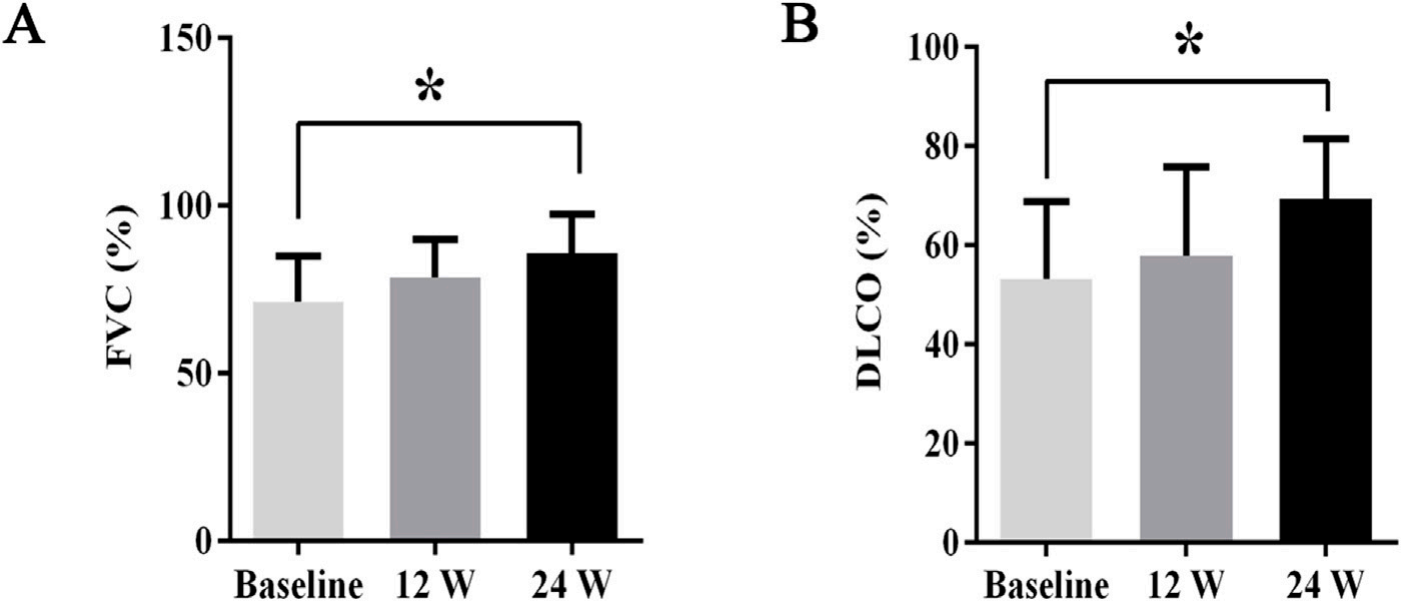
The changes in FVC% and DLCO% before and after treatment. Post-treatment, **(A)** FVC% increased by 20.4%, **(B)** DLCO% improved by 30.2%. All data are presented as means ± standard errors of the means, with *p < 0.05 indicating statistical significance compared to pretreatment data.

During treatment, all patients were monitored for 24 weeks. Over the course of this treatment, prednisone acetate dosage was gradually reduced to 5–7.5 mg/day, whereas that of telitacicept was increased to 160 mg every 2 weeks. No patient experienced muscle pain, weakness, rash, or joint pain, and all reported considerable relief from dyspnea. Laboratory indicators, including CK, LDH, ALT, AST, ferritin, ESR, and CRP, returned to normal levels (Table 1). The PaO2/FiO2 ratio improved by 28.2% compared with baseline (Figure 1A), whereas the 6MWT outcomes improved by 34% (Figure 1C). In all patients with muscle involvement, the MMT-8 score increased by 25.1% (Figure 1B). All patients’ chest HRCT scans revealed varying degrees of reduction in bilateral lung lesions (Figure 2), and lung function demonstrated considerable improvements (Figure 3); in particular, compared with baseline, FVC% and DLCO% increased by 20.4% and 30.2%, respectively (Figure 4). Only one patient received additional treatment with nintedanib for pulmonary fibrosis due to insufficient improvement in lung function.

Regarding adverse events, one patient experienced a mild lung infection; nevertheless, no further infection occurred after telitacicept dosage was reduced. Two patients experienced pruritus at the injection site, lasting for 1–2 days, which resolved spontaneously. None of the patients exhibited leukopenia, gastrointestinal disorders, or neurological diseases; moreover, they demonstrated minimal IgG-related adverse reactions during treatment, indicating good safety.

## 4 Discussion

The current recommendations for IIM-ILD treatment have been primarily based on empirical research; moreover, definitive guidelines for the optimal treatment of IIM-ILD remain unavailable. Recommendations suggest that treatment decisions should be tailored to a patient’s specific form of ILD and myositis antibody profile (Fujisawa, 2021). For patients with acute and subacute ILD, including those with anti-MDA5 antibody-positive DM and ASS, high-dose GCs should be combined with CNIs (Fujisawa, 2021; Fujisawa et al., 2021; Chen et al., 2022). A triple therapy regimen comprising GCs, CNIs, and CTX may be administered to patients with rapid ILD progression and multiple adverse prognostic factors (Tsuji et al., 2020; Fujisawa, 2021). For patients with refractory ILD, options such as RTX, plasma exchange, and tofacitinib may be considered. Additionally, intravenous immunoglobulin has the functions of regulating immune cell activity, neutralizing autoantibodies, and downregulating chemotactic cytokines. Therefore, for patients with recurrent or refractory IIMs-ILD, intravenous immunoglobulin therapy can be administered (Gandiga et al., 2023). For those with chronic ILD, GCs and immunosuppressants, including CTX, azathioprine, MMF, and CNIs, may be administered (Mecoli and Christopher-Stine, 2018; Fujisawa, 2021; Moda et al., 2025).

IIM-ILD pathogenesis remains unclear. Nevertheless, in locally inflamed tissue of patients with DM, B-cell infiltration occurs around blood vessels and the muscle endometrium (Saketkoo et al., 2010). Moreover, JO-1 antibodies have recently been isolated from the alveolar lavage fluid of patients with IIM-ILD (Galindo-Feria et al., 2020). Activated B cells differentiate into plasma cells, which produce various antibodies such as antisynthetase and MDA5 antibodies; these antibodies bind to lung autoantigens and trigger tissue damage (Moda et al., 2025). Furthermore, studies have reported that follicular dendritic, T, and B cells contribute to the formation of inducible bronchus-associated lymphoid tissue (iBALT) at pulmonary interstitial lesion sites. iBALT exhibits germinal center activity, facilitating the induction of plasma cells that produce high-affinity antibodies, release neutrophil extracellular traps, and contribute to pulmonary fibrosis development (Akiyama and Kaneko, 2022). These findings indicate that B cells and plasma cells may be pivotal in IIM-ILD pathogenesis.

RTX, a chimeric CD20 monoclonal antibody, targets B cells and induces B-cell depletion; it is thus used in salvage treatment for refractory ILD (Reff et al., 1994; Ogawa et al., 2017; Liossis and Bounia, 2022). RTX exhibits substantial efficacy in patients with refractory ASS-ILD, severe ASS-ILD, recurrent or progressive ASS-ILD, and clinically amyopathic DM characterized by rapidly progressive (RP) ILD, as well as in patients with MDA5 antibody-positive DM-ILD (Allenbach et al., 2015; Doyle et al., 2018; So et al., 2018; Ge et al., 2021). However, RTX treatment can increase B-lymphocyte stimulator (BLyS) levels, possibly promoting reemergence of autoreactive B cells and contributing to disease recurrence over time (Cambridge et al., 2008; Ehrenstein and Wing, 2016).

BLyS, a type II membrane-bound protein from the tumor necrosis factor family, is mainly secreted by monocytes and macrophages (Mockel et al., 2021). BLyS is essential for B-cell survival, maturation, and class-switching, as well as plasma cell survival (Vincent et al., 2013). It also plays a major role in the pathogeneses of various autoimmune diseases. Patients with DM exhibiting elevated BLyS levels have an increased ILD risk (Matsushita et al., 2019). Furthermore, serum BLyS levels are correlated with ILD severity (Hamada et al., 2015). Notably, serum BLyS levels are positively associated with rapidly progressive ILD (RP-ILD) occurrence in patients with MDA5-positive DM (Shi et al., 2024). Therefore, BLyS may be strongly associated with IIM-ILD pathogenesis.

Telitacicept is a novel recombinant fusion protein, comprising the ligand-binding domain of the TACI receptor and the Fc segment of human IgG. It can effectively bind to and inhibit BLyS and proliferation-inducing ligand (APRIL) activities, thereby suppressing the development and survival of plasma cells and mature B cells (Dhillon, 2021; Shi et al., 2021; Fan et al., 2022). This drug has recently demonstrated favorable clinical efficacy and safety in Sjögren’s syndrome (Xu et al., 2024), systemic lupus erythematosus (SLE) (Jin et al., 2023; Wu et al., 2024), and rheumatoid arthritis (RA) (Dhillon, 2021). However, its therapeutic effect on IIM-ILD has not been reported thus far.

A combination of RTX and telitacicept can effectively neutralize elevations in BLyS levels, thereby delaying B-cell reactivation, improving remission rates, and reducing recurrence rates in patients with refractory lupus nephritis (Chen et al., 2025a; 2025b). Furthermore, sequential treatment with RTX and telitacicept can significantly reduce recurrence rates in patients with refractory anti-NMDA receptor encephalitis and MOG-associated demyelination (Zhang et al., 2025). However, the therapeutic effects of this combination in IIM-ILD have not yet been reported.

In the current study, we included 10 IIM-ILD cases; of them, four, who were newly diagnosed as having severe ILD, were treated with a combination of RTX and telitacicept. Six patients exhibited refractory IIM-ILD. Of them, four were treated with telitacicept because they had a high risk of infection associated with RTX; the remaining two patients were administered a combination of RTX and telitacicept. All 10 patients demonstrated symptom relief and considerable improvements in laboratory and imaging indicators, thereby providing the dual treatment effects for idiopathic inflammatory IIM-ILD. Telitacicept demonstrated favorable clinical efficacy and safety in our patients with anti-MDA5 antibody-positive DM-associated ILD and ASS-ILD.

Our results indicated that telitacicept can be used as a novel therapeutic for IIM-ILD treatment. However, because we did not include a control group, our results and conclusions may be constrained. Future studies should employ a prospective, controlled design to further validate the efficacy of telitacicept, both alone and in combination with RTX, in managing IIM-ILD. In addition, further extensive multicenter clinical studies are necessary to confirm the safety and efficacy of telitacicept in patients with connective tissue diseases and interstitial lung lesions, such as Sjögren’s syndrome, systemic sclerosis, and antineutrophil cytoplasmic antibody–associated vasculitis.

## Data Availability

The original contributions presented in the study are included in the article/supplementary material, further inquiries can be directed to the corresponding author.
